# Amelioration of injury-induced tissue acidosis by a nonsteroidal analgesic attenuates antinociceptive effects of the pH-dependent opioid agonist NFEPP

**DOI:** 10.1038/s41598-022-19568-9

**Published:** 2022-09-07

**Authors:** Melih Ö. Celik, Roger Negrete, Riccardo Di Rosso, Halina Machelska, Christoph Stein

**Affiliations:** Department of Experimental Anesthesiology (CBF), Charité-Universitätsmedizin Berlin, Campus Benjamin Franklin, Hindenburgdamm 30, 12203 Berlin, Germany

**Keywords:** Drug discovery, Neuroscience

## Abstract

Opioid agonists are powerful drugs for managing pain. However, their central side effects are limiting their use and drugs with similar potency, but a lower risk profile are needed. (±)-N-(3-fluoro-1-phenethylpiperidine-4-yl)-N-phenylpropionamide (NFEPP) is a novel opioid agonist that preferentially activates opioid receptors at acidic extracellular pH. NFEPP was designed to activate peripheral opioid receptors in injured tissue, therefore precluding side effects elicited at normal pH in brain or intestinal wall. Considering the common combination of opioids and nonsteroidal anti-inflammatory drugs (NSAIDs) in multimodal analgesia, we investigated the interaction between NFEPP and a widely prescribed prototypical NSAID, diclofenac (DCF), in a rat model of unilateral hindpaw inflammation induced by complete Freund’s adjuvant. We evaluated the effects of systemically applied DCF on the paw tissue pH, on the expression of inflammatory mediators in immune cells from inflamed paws and on the expression of opioid receptors in dorsal root ganglia. Additionally, we investigated the antinociceptive efficacy of NFEPP injected into the inflamed paws after DCF treatment. We found that DCF reduced inflammation-induced nociceptive responses and tissue acidosis, but did not change the mRNA expression of IL-1β, TNF-α, IL-6, IL-4, NGF, or of mu-, delta-, or kappa-opioid receptors. The treatment with DCF moderately reduced the antinociceptive efficacy of NFEPP, suggesting a correlation between an increase in local tissue pH and the decreased antinociceptive effect of this pH-sensitive opioid agonist.

## Introduction

Opioids are the strongest painkillers and they are commonly used for severe pain after surgery, trauma or associated with cancer; yet, their application is limited due to their severe side effects including addiction, respiratory depression and constipation. Misuse of both prescription and non-prescription opioids have dramatically increased worldwide^1^.

An alternative approach to pain control is to target opioid receptors on peripheral sensory neurons, thereby avoiding central adverse effects^2^. (±)-N-(3-fluoro-1-phenethylpiperidine-4-yl)-N-phenylpropionamide (NFEPP) has a low acid dissociation constant (pK_a_ = 6.8), and preferentially activates mu-opioid receptors (MOR) in acidotic injured tissue without activating MORs in healthy tissues such as brain or intestinal wall^3–5^.

Non-opioid based medications including nonsteroidal anti-inflammatory drugs (NSAIDs) such as diclofenac (DCF), are also widely used for pain management; however, these substances are limited by their weaker analgesic properties and by cardiovascular, gastrointestinal and renal side effects^6^. To maximize pain relief while minimizing adverse effects, the concomitant administration of NSAIDs and opioid agonists is advised^7,8^. In this study, we evaluated the interactions between NFEPP and DCF in a rat model of inflammatory pain induced by complete Freund’s adjuvant (CFA) injected unilaterally into the rat hindpaw. We also tested fentanyl, a commonly used opioid, as a control for NFEEP. In addition to antinociception, we assessed the effects of DCF on the paw tissue pH, on the expression of inflammatory mediators in immune cells isolated from inflamed paws and on the expression of opioid receptors in dorsal root ganglia (DRG).

## Results

### CFA induces inflammation and mechanical hypersensitivity, and decreases pH at the inflammation site

**Figure 1:**
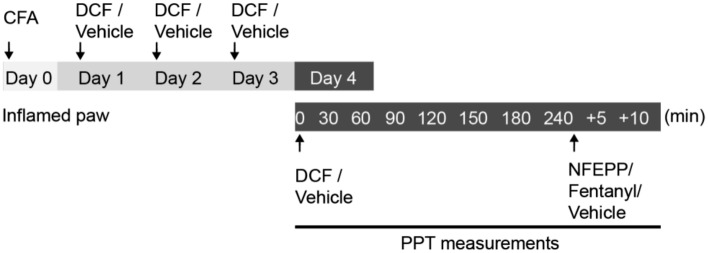
Experimental protocol. Complete Freund's adjuvant (CFA) was injected intraplantarly (i.pl.) into the right hindpaw on day 0. Diclofenac (DCF) or vehicle were injected subcutaneously (s.c.) daily for 4 days starting 24 h after CFA (day 1). The paw pressure threshold (PPT) was assessed 4 days after CFA before (0 min) and 30–240 min after the last DCF/vehicle application. After that, NFEPP, fentanyl or vehicle were administered i.pl. into the inflamed paw and PPT was measured again 5 and 10 min later (+ 5 and + 10).

**Figure 2:**
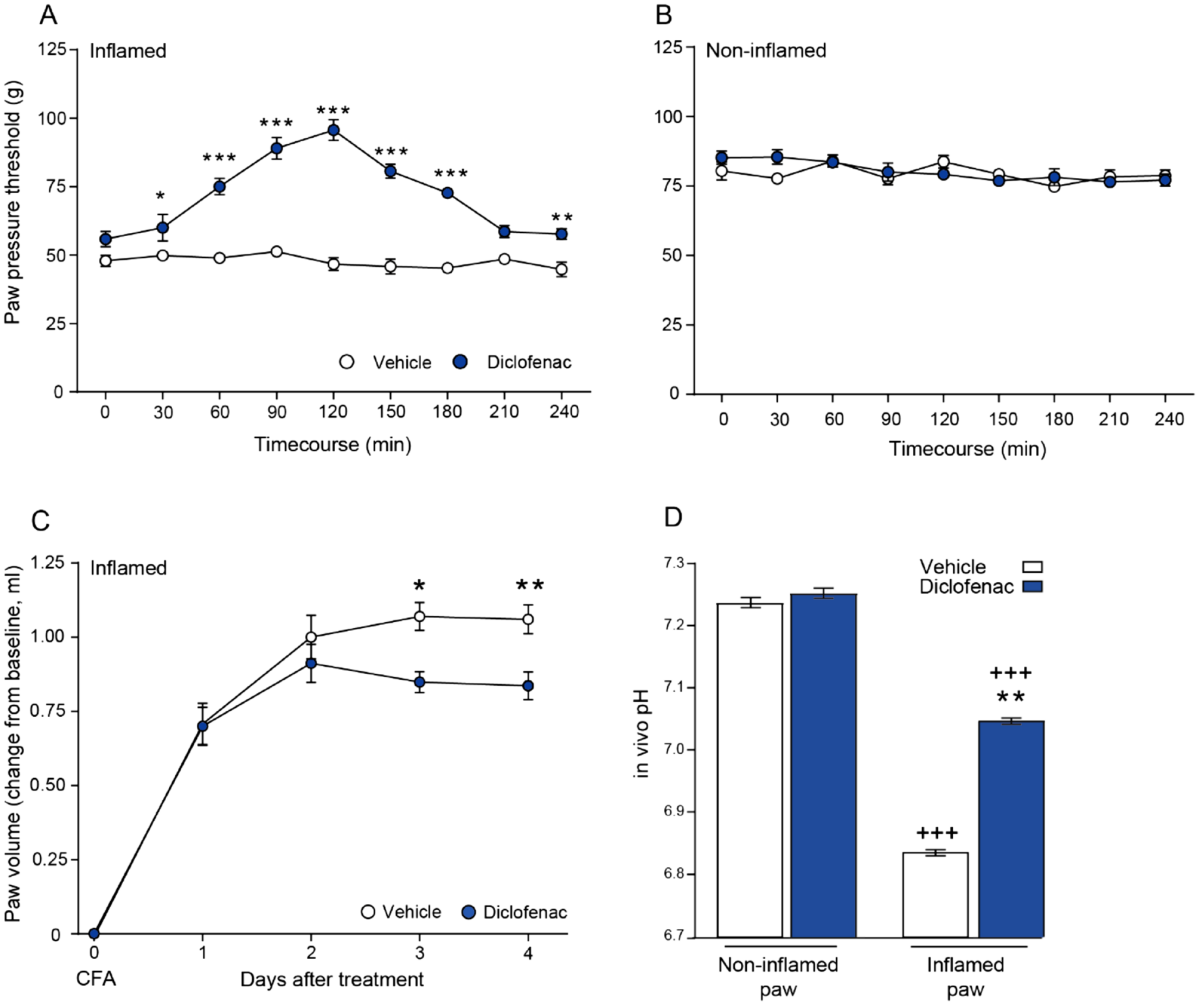
Effects of subcutaneous (s.c.) diclofenac on nociceptive responses, inflammation and tissue pH. The paw pressure threshold (PPT) values after daily s.c. diclofenac (DCF; 20 mg/kg) injections in inflamed (**A**) and in contralateral non-inflamed (**B**) hindpaws, assessed at 4 days after intraplantar (i.pl.) complete Freund's adjuvant (CFA) inoculation before (0) and up to 240 min after the last DCF injection. *P < 0.05, **P < 0.01, ***P < 0.001 DCF versus vehicle, two-way RM ANOVA and Bonferroni tests. (**C**) Decrease of inflamed paw volume after daily s.c. DCF (20 mg/kg) injection measured for 4 days after i.pl. CFA inoculation after each DCF injection. *P < 0.05, **P < 0.01 DCF versus vehicle, two-way RM ANOVA and Bonferroni test. (**D**) pH values in inflamed paw tissue after DCF treatment (20 mg/kg s.c.) at 4 days after i.pl. CFA inoculation. **P < 0.01 DCF versus vehicle, two-tailed unpaired t-test; ^+++^*P* < 0.001 inflamed versus non-inflamed paw, two-tailed paired t-test. Data show means ± SEM (n = 8 rats per group).

To induce hindpaw inflammation, we injected intraplantarly (i.pl.) CFA. Four days after injection (Fig. 1), we observed intense mechanical hypersensitivity, manifested by reduced paw pressure thresholds (PPT) in inflamed paws compared to contralateral non-inflamed paws (47.91 g vs. 80.41 g, respectively; *P* < 0.001) (Fig. 2A, Fig. 2B). The volume of the inflamed hindpaws was significantly increased (1.06 ml vs. 0.83 ml, inflamed vs. contralateral non-inflamed paws; *P* < 0.01) (Fig. 2C). Additionally, the tissue pH in the i.pl. CFA injected paws was significantly lower when compared to non-inflamed contralateral paws (6.84 vs. 7.24, respectively; *P* < 0.001) (Fig. 2D).

### Diclofenac treatment decreases hyperalgesia, paw volume and tissue acidosis

To investigate the anti-inflammatory effects of diclofenac treatment in inflamed paws, we injected diclofenac (20 mg/kg; s.c.) daily, for 4 days following CFA injection. DCF treatment attenuated CFA-induced mechanical hypersensitivity, as measured by increased PPT in the inflamed paws. The effect peaked at 120 min after the last DCF injection and returned to the pre-injection (0 min) levels at 210–240 min. Vehicle administration did not alter PPT (Fig. 2A). There were no changes in PPT in the contralateral non-inflamed paws after DCF treatment (Fig. 2B). The CFA-induced increase in paw volume was also attenuated on days 3 and 4 after DCF injection (Fig. 2C). At 4 days after CFA, DCF significantly increased pH in the inflamed paw, but did not alter pH in the contralateral non-inflamed paws (Fig. 2D).

### Diclofenac treatment decreases the antinociceptive efficacy of NFEPP

**Figure 3:**
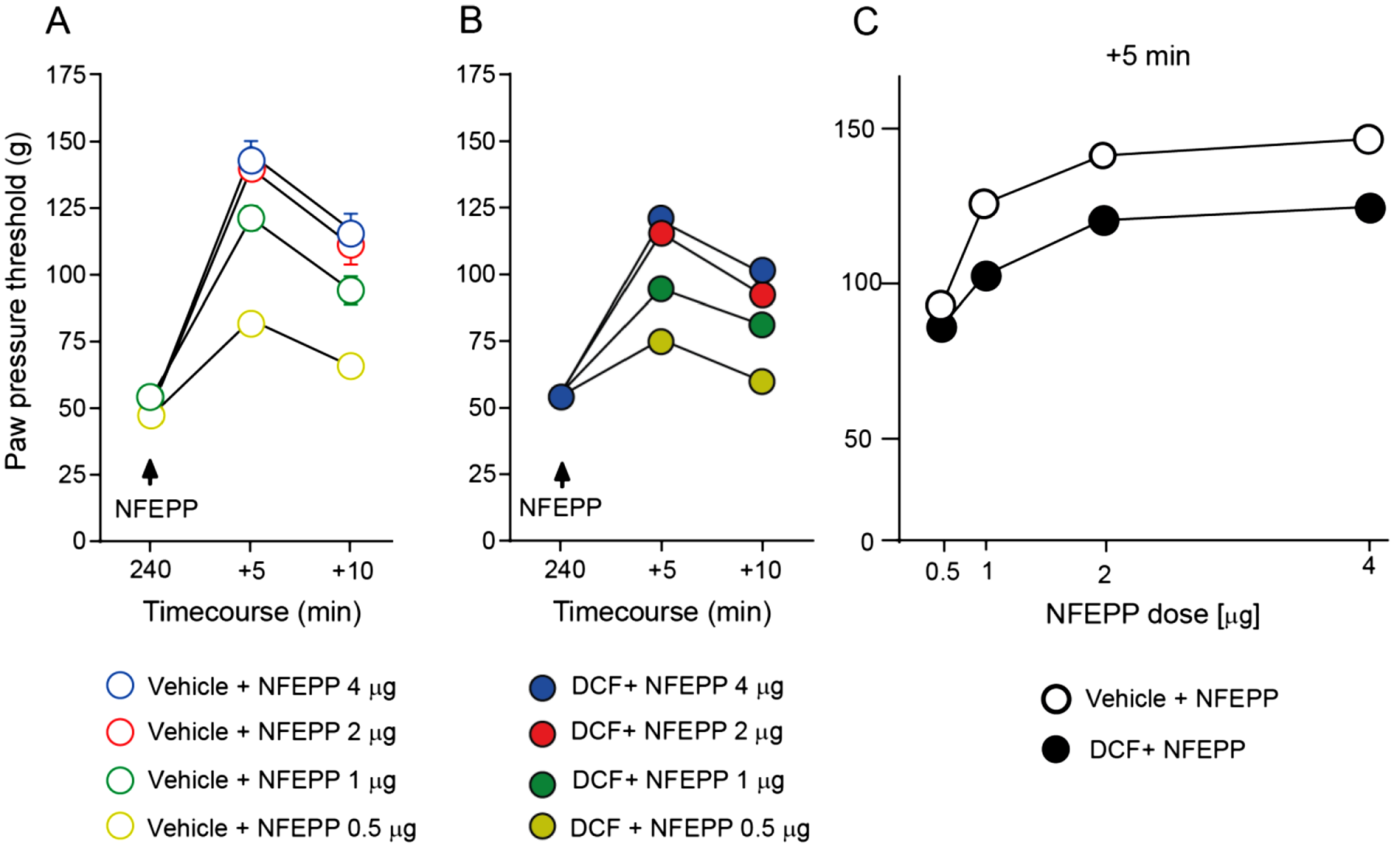
Antinociceptive efficacy of intraplantar (i.pl.) NFEPP after subcutaneous (s.c.) diclofenac. Dose- and time-dependent paw pressure threshold (PPT) elevations in inflamed paws after NFEPP (0.5–4 µg) injected i.pl. into inflamed paws, assessed after s.c. treatment with vehicle (**A**) or diclofenac (DCF; 20 mg/kg) (**B**) at 4 days after i.pl. complete Freund's adjuvant inoculation. All PPT elevations were statistically significant at + 5 min (*P* < 0.05 vs. 240 min, one-way RM ANOVA and Bonferroni test; not indicated for graph clarity). (**C**) DCF (20 mg/kg; s.c. daily for 4 days)-induced attenuation of PPT elevation produced by increasing doses of NFEPP (i.pl.) in inflamed paws at 5 min after NFEPP injection. *P* < 0.01 versus vehicle, area under the curve, two-tailed unpaired t-test. Data show means ± SEM (n = 8 rats per group).

To assess the antinociceptive effects of NFEPP in in the CFA-induced inflammation, NFEPP (0.5–4 µg) was injected i.pl. into inflamed paws of rats pretreated with s.c. vehicle (NaCl). NFEPP elevated PPT of the inflamed paws for the following 10 min, with a peak effect at 5 min (Fig. 3A). There were no changes in PPT in the contralateral non-inflamed paws (*P* > 0.05, not shown). To evaluate if DCF treatment influenced the antinociceptive efficacy of NFEPP, we injected i.pl. NFEPP into inflamed paws on day 4 at 240 min after the last s.c. DCF injection, i.e. when the DCF-induced PPT elevation had dissipated (Figs. 1, 2A). The antinociceptive effects of all 4 dosages of NFEPP were attenuated when DCF was injected prior treatment (Fig. 3B compared to Fig. 3A). For clarity of presentation, only NFEPP doses at + 5 min (most effective NFEPP antinociception ) are shown in Fig. 3C (area under the curve, total area: 119 vs. 203 DCF + NFEPP vs. vehicle + NFEPP; *P* < 0.01). There were no changes in PPT in contralateral non-inflamed paws after DCF or NFEPP treatment (*P* > 0.05, not shown).

### Diclofenac treatment does not influence the antinociceptive efficacy of fentanyl

**Figure 4:**
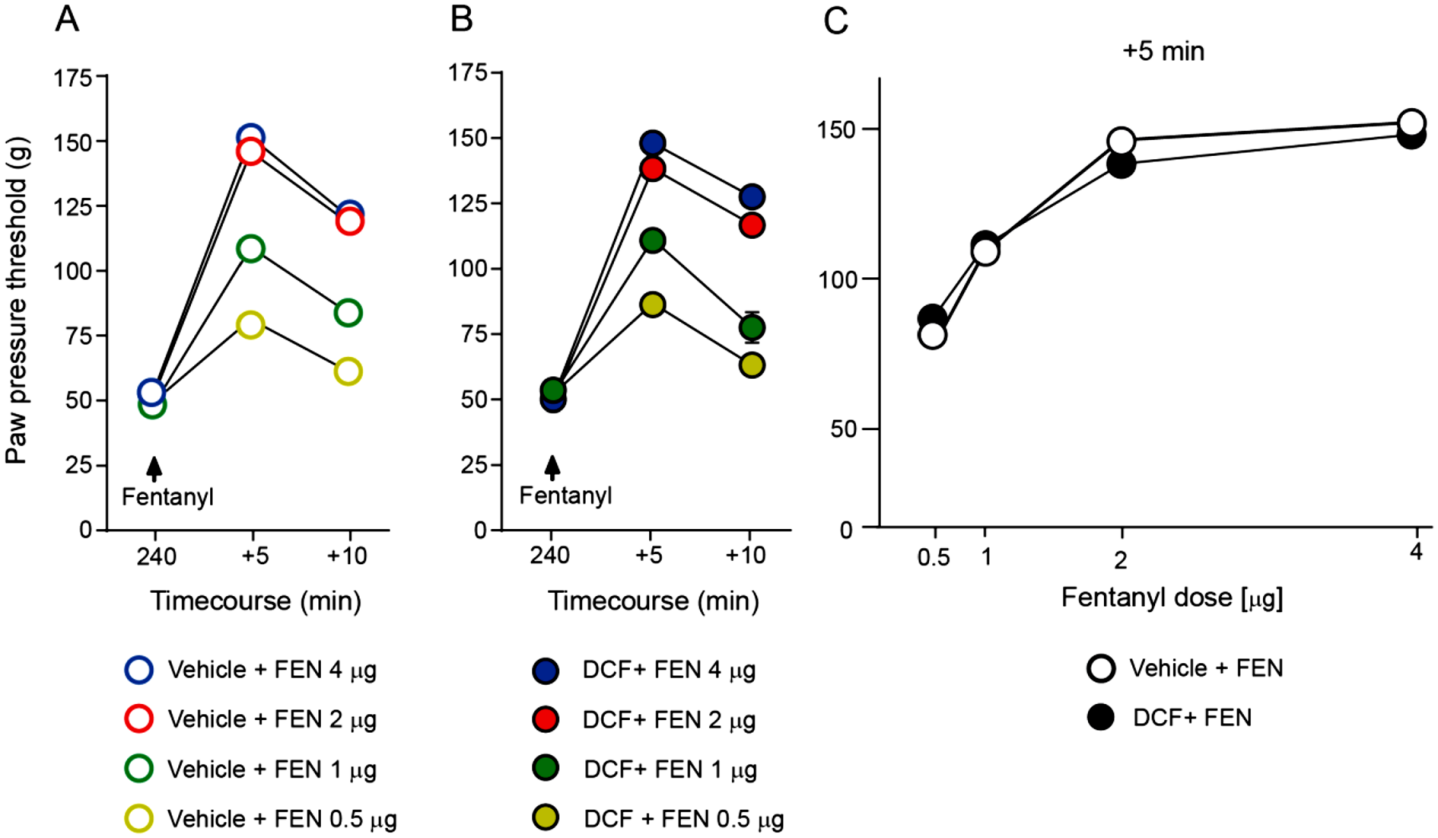
Antinociceptive efficacy of intraplantar (i.pl.) fentanyl after subcutaneous (s.c.) diclofenac. Dose- and time-dependent paw pressure threshold (PPT) elevations in inflamed paws after fentanyl (0.5–4 µg) injected i.pl. into inflamed paws, assessed after daily s.c. treatment with vehicle (**A**) or diclofenac (DCF; 20 mg/kg) (**B**) at 4 days after i.pl. complete Freund's adjuvant inoculation. All PPT elevations were statistically significant (*P* < 0.05 vs. 240 min, one-way RM ANOVA and Bonferroni test; not indicated for graph clarity). (**C**) PPT elevations produced by increasing doses of fentanyl (i.pl.) in inflamed paws at 5 min after fentanyl injection were not different between vehicle- and DCF-pretreated groups. *P* > 0.05 versus vehicle, area under the curve, two-tailed unpaired t-test. Data show means ± SEM (n = 8 rats per group).

Next, we used fentanyl, a commonly used MOR agonist, as a control to assess if the DCF induced decrease in NFEPP efficacy was agonist-specific. In rats pretreated with s.c. vehicle (NaCl), fentanyl (0.5–4 µg) injected i.pl. into inflamed paws elevated PPT in these paws for the following 10 min, with a peak effect at 5 min (similar to NFEPP) (Fig. 4A). The PPT in contralateral paws did not change at low doses (0.5 and 1 μg; *P* > 0.05 vs. 240 min) and increased at higher doses of fentanyl (2 and 4 µg) at 5 min (*P* < 0.001 vs. 240 min) (data not shown). On day 4 and 240 min after the last DCF injection (20 mg/kg s.c.), the antinociceptive efficacy of i.pl. fentanyl was not influenced by this pretreatment (Fig. 4B,C). For clarity of presentation, only fentanyl doses at + 5 min are shown in Fig. 4C (area under the curve, total area 198 vs. 210, DCF + fentanyl vs. vehicle + fentanyl; *P* > 0.05).

### Diclofenac does not change the mRNA expression of inflammatory markers in the inflamed paw

**Figure 5:**
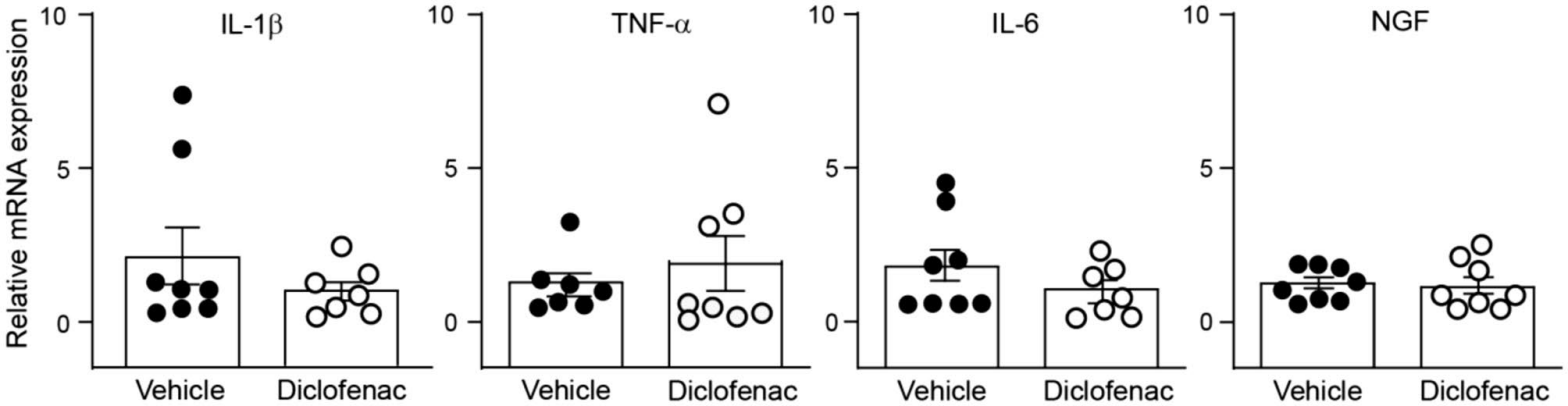
Expression of inflammatory mediator mRNAs in inflamed paw tissue. mRNA levels of IL-1β, IL-6, TNF-α and NGF after daily subcutaneous treatment with vehicle or diclofenac (DCF; 20 mg/kg) at 4 days after intraplantar complete Freund's adjuvant inoculation. *P* > 0.05 versus vehicle, two-tailed unpaired t-test. Data represent relative mRNA expression levels normalized to GAPDH, are expressed as fold change versus vehicle, and are shown as means ± SEM and individual data points (n = 7–8 samples per group).

Additionally, we tested the effects of diclofenac on the mRNA expression of inflammatory markers in immune cells isolated from the inflamed paws at 4 days after CFA. The mRNA expression of IL-1β, IL-6, TNF-α and NGF did not change after DCF treatment (20 mg/kg s.c.) compared to cells isolated from s.c. vehicle-treated control animals (Fig. 5). Because we have previously shown that IL-4 induced the release of opioid peptides from macrophages^22^, we also attempted to examine the mRNA of IL-4, but did not detect it in immune cells from inflamed paws of either DCF- or vehicle-treated control animals (data not shown).

### Diclofenac treatment does not change the mRNA expression of opioid receptors in DRG

**Figure 6:**
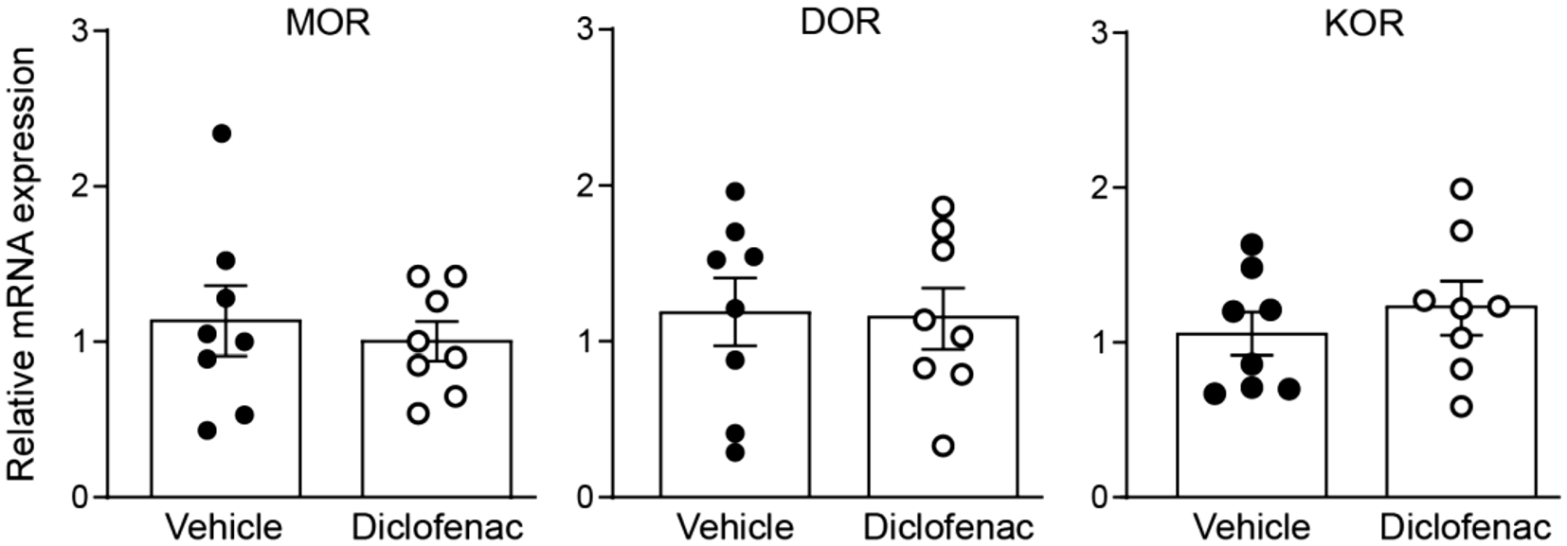
Expression of opioid receptor mRNAs in DRG. mRNA levels of MOR, DOR and KOR after daily subcutaneous treatment with vehicle or diclofenac (DCF; 20 mg/kg) at 4 days after intraplantar complete Freund's adjuvant inoculation. *P* > 0.05 versus vehicle, two-tailed unpaired t-test. Data represent relative mRNA expression levels normalized to GAPDH, are expressed as fold change versus vehicle, and are shown as means ± SEM and individual data points (n = 8 samples per group).

To evaluate whether the decreased antinociceptive efficacy of i.pl. NFEPP after s.c. DCF treatment might be due to changes in the expression of opioid receptors in peripheral sensory neurons innervating the inflamed tissue, we examined the mRNA expression of MOR, delta-opioid receptors (DOR) and kappa-opioid receptors (KOR) in DRG (L4-L6). The qRT-PCR data revealed no changes after DCF (20 mg/kg) treatment compared to control animals (Fig. 6).

## Discussion

The main goal of this study was to investigate interactions between the novel opioid agonist NFEPP and DCF, a typical NSAID that is expected to modulate inflammation and associated tissue acidosis^9^. Previous studies showed the ability of NFEPP to produce antinociception in models of inflammatory, neuropathic or visceral pain without exerting typical side effects of conventional opioid agonists^3,4,10–13^. In the past, different approaches to avoid opioid side effects were pursued (reviewed in^5^). One strategy is to restrict analgesic activity to peripheral tissue, for example based on the reduction of blood brain barrier permeability of compounds^14^. This was achieved by chemical modification of opioid agonists, e.g. by linking with larger molecules or synthesis of peptide-based compounds^1,15,16^. The recognition of the paramount role of protonation in the ability of opioid agonists to bind and activate opioid receptors is the basis of the design of NFEPP and its property to not exert central side effects^4^.

It is important to investigate interactions between NFEPP and DCF as both drugs are designed for the treatment of inflammatory pain and because co-administration of NSAIDs and opioids in multimodal analgesia is nowadays considered a standard approach in pain therapy. In our inflammatory pain model, pretreatment with DCF reduced the antinociceptive efficacy of acutely administered NFEPP, albeit by only about 18%. This reduction was present at NFEPP dosages that were known to exert antinociception without causing typical opioid central side effects in rats^4^. We hypothesized that, if there was an increase of pH in inflamed tissue following the administration of an anti-inflammatory drug, this could be the reason behind the reduction of NFEPP’s antinociceptive efficacy. Therefore, it was first necessary to verify that DCF was able to effectively reduce inflammation and local acidosis. The importance of this aspect is illustrated by the peculiar pharmacodynamics of NFEPP: its functional restriction to opioid receptors expressed in peripheral inflamed tissue is due to its reduced pK_a_ (6.8) and consequently to its selective protonation in acidotic environments^11,13^. In contrast, the conventional opioid fentanyl has a pK_a_ value of 8.4^17^ and is therefore protonated at both normal and acidotic pH values. As expected, DCF did not influence the antinociceptive efficacy of fentanyl.

To exclude other confounding factors, we evaluated inflammatory mediators that could have an influence on nociceptive responses measured in our study. The qRT-PCR experiments did not show any differences in the expression of IL-1β, IL-6, TNF-α or NGF mRNAs between vehicle- and DCF-treated animals. The mRNAs of these inflammatory mediators were detectable at similar levels in rats treated with DCF and in those belonging to the control group, and IL-4 mRNA was expressed neither in the former nor in the latter. Thus, the reduced antinociceptive efficacy of NFEPP cannot be ascribed to an effect of the NSAID on the above mentioned mediators.

Another variable that we wanted to exclude as confounding factor was a possible difference in opioid receptor expression in DRG. Inflammation of peripheral tissue is known to affect expression of opioid receptors in DRG^18^. NFEPP is an opioid receptor agonist^4^ and therefore, there was a possibility that the reduction of its efficacy could be due to changes in the regulation of opioid receptor expression. However, there was no difference in expression of genes coding for MOR, DOR and KOR between rats treated with DCF or vehicle, showing that the treatment with the NSAID had no influence on opioid receptor expression in DRG. In line with these findings, the efficacy of fentanyl was not different between controls and animals pretreated with DCF.

In summary, our study showed that treatment with an NSAID is able to increase the pH of inflamed tissue, and that this modification is correlated with a moderate reduction of the peripheral antinociceptive efficacy of NFEPP. We did not find any modification of the expression of inflammatory mediators or opioid receptors that could have provided an alternative explanation for this observation. This confirms the previously shown pH-dependency of NFEPP^3,4,13^. Although these findings suggest that combining NFEPP with NSAIDs may not be advantageous, ideally, using peripherally acting opioids without central adverse effects might reduce the need to use multimodal analgesia, eventually decreasing drug interactions and the potential for additional side effects.

## Methods

### Animals

Male Wistar rats (200–300 g, 6–7 weeks old) were purchased from Janvier Laboratories (Les Boudieres, France). The rats were kept on a 12-h light/dark schedule, in groups of 2 in cages lined with ground corncob bedding, with free access to standard laboratory food and water. Room temperature was 22 ± 0.5 °C and humidity was 60–65%. Animals were randomly placed in cages by an animal caretaker who was not involved in the study. Before nociceptive testing, handling was performed once per day for 3 days*. *In vivo behavioral experiments and pH measurements were performed by an investigator blinded to the treatments and drug doses. Similarly, in vitro experiments were performed by an investigator blinded to the sample assignments by coded vials. The codes were broken after the completion of the experiments. Experimental protocols were approved by the state animal ethics committee (Landesamt für Gesundheit und Soziales, Berlin, Germany) and were performed following the recommendations of the International Association for the Study of Pain and the ARRIVE guidelines^19^. Statistical power calculations were conducted to determine the group sizes and every effort was made to minimize the animals’ suffering.

### Chemicals and drugs

Diclofenac sodium (2-[2-(2,6-dichloroanilino)phenyl]acetic acid; sodium) and fentanyl citrate (N-phenyl-N-[1-(2-phenylethyl)-4-piperidinyl] propanamide citrate salt) were purchased from Sigma Aldrich (Darmstadt, Germany). NFEPP was synthesized according to our design by a contractor (ASCA GmbH, Berlin, Germany)^4^. Isoflurane was purchased from AbbVie (Ludwigshafen, Germany) and CFA was purchased from Calbiochem (La Jolla, CA, USA). NFEPP was dissolved in dimethyl sulfoxide (0.5% DMSO), fentanyl and DCF were dissolved in water, and all compounds were diluted with 0.9% NaCl to obtain the final concentrations. Control animals received 0.9% NaCl. TRIzol Reagent was purchased from Thermo Fisher Scientific (Waltham, MA, USA). Digestive solution was prepared using 10 ml RPMI with GlutaMax, 0.5 ml of HEPES (both from Thermo Fischer Scientific, Waltham, MA, USA), 30 mg of collagenase, 10 mg of hyaluronidase, 200 µl of FBS 2% (all from Sigma-Aldrich, Darmstadt, Germany). FACS buffer was prepared using 500 ml PBS (Sigma-Aldrich, Darmstadt, Germany) and 10 ml FBS 2%.

### Paw inflammation

Rats received an intraplantar (i.pl.) injection of CFA (150 µl) into the right hindpaw under brief isoflurane anesthesia. Behavioral tests were performed 4 days after CFA administration, as described previously^2,11,12,14^.

### Drug injections

Rats received daily subcutaneous (s.c.; into the skin fold on the back) DCF (20 mg/kg in 200 µl) or vehicle (200 µl) injections for 4 days starting 24 h after the induction of inflammation to assess whether DCF alone reduced nociceptive responses, inflammation or tissue acidosis. On day 4, 240 min after s.c. DCF or vehicle, rats were additionally injected i.pl. into the inflamed paw with NFEPP (0.5–4 µg in 100 µl), fentanyl (0.5–4 µg in 100 µl) or vehicle (100 µl) (Fig. 1). All dosages were determined in pilot experiments.

### Mechanical sensitivity (Randall-Selitto test)

Rats were gently held under paper wadding and incremental pressure was applied via a wedge-shaped, blunt piston onto the dorsal surface of the hindpaws using an automated gauge (Ugo Basile, Comerio, Italy). The paw pressure threshold (PPT) required to induce paw withdrawal was determined by averaging three consecutive trials separated by 15 s intervals. Hypersensitive values of PPT usually ranged between 30–60 g. Normal baselines usually ranged between 70 and 90 g. The cut-off was set at 250 g to avoid tissue damage. The sequence of paws was alternated between animals to avoid order effects^2,11,14,15^. The measurements were performed on day 4 after induction of inflammation before (0 min) and 30, 60, 120 and 240 min after the injection of DCF/vehicle. After that, rats additionally received i.pl. NFEPP, fentanyl or vehicle. PPT was measured again 5 and 10 min later (denoted as “+ 5” and “+ 10”; Fig. 1).

### Paw volume measurement

Anti-inflammatory effects of DCF were assessed by monitoring paw volume (PV) each day with a plethysmometer (Ugo Basile, Comerio, Italy). The volume of displacement, which is equal to the PV, was indicated on a digital display^2,14^. The difference between inflamed and non-inflamed paws was determined.

### In vivo pH measurements

On day 4, after the 4th DCF injection, rats were anesthetized with isoflurane using a Datex-Ohmeda Aestiva 3000 anesthesia machine while spontaneous breathing was maintained. A pH glass microelectrode (VWR, Hamden, USA) was calibrated using reference solutions of pH 4.0, 7.0 and 9.2. The microelectrode was mounted into a 20 G needle, and then inserted into the plantar side of the hindpaw. After obtaining a stable signal during the first 5 min, pH values were recorded every 20 s during the next 5 min, and the mean of 15 pH values were calculated^13,20^.

### Dorsal root ganglion (DRG) isolation

On day 4, after the 4th DCF injection, the DRG (L4, L5 and L6) sensing the right hindpaw were extracted to isolate RNA. The spinal cord was carefully removed using watchmaker forceps, and DRG were exposed. Rib bones were used as anatomical landmarks to identify L4, L5 and L6 DRG, which were then extracted using curved watchmaker forceps. The three DRG were pooled in a screw cap 1.5 ml tube, deep frozen in liquid nitrogen and stored at − 80 °C until RNA extraction^21,22^.

### Isolation of subcutaneous tissue from inflamed paws

On day 4, after the 4th DCF injection, subcutaneous tissue from the plantar side of inflamed hindpaws was collected and digested using digestive solution to isolate cells. All tissue more superficial than the plantar fascia was isolated, hashed and put in 2 ml of the digestive solution. Rats were sacrificed by cervical vertebral fracture under general anesthesia with isoflurane.

### Isolation of immune cells from subcutaneous tissue

The tube containing tissue in digestive solution, was incubated at 37 °C for 60 min. The solution was then filtered through a 70 µm pore sieve, using 30 ml of FACS buffer during filtration. The tube was centrifuged for 5 min at 380*g* and 4 °C, the supernatant was discarded, the pellet re-suspended in 4 ml erythrocyte lysis buffer (Sigma-Aldrich, Darmstadt, Germany), mixed well and incubated on ice for 4 min. 20 ml of FACS buffer were then added to the solution, centrifuged at 350*g* and 4 °C for 5 min, and the supernatant was discarded. The pellet was re-suspended in 1 ml of FACS buffer. 1 µl was used to assess cell number and viability by a dual fluorescence cell counter (Logos Biosystems, Villeneuve-d'Ascq, France), and the remaining material was stored at − 80 °C until RNA extraction^21,22^.

### RNA isolation from the DRG

DRG (L4, L5 and L6) from 2 rats, ipsilateral to inflamed paws were pooled to obtain a total amount of 6 DGR per sample. DRG were pulverized in liquid nitrogen using mortar and pestle, transferred to a tube with the addition of 1 ml TRIzol reagent, briefly vortexed and stored for 30 min at 4 °C. The solution was then centrifuged for 5 min at 350*g* and 4 °C, supernatant was transferred to a phase-maker tube (Thermo Fischer Scientific, Waltham, MA, USA) and incubated in room temperature for 5 min. 0.2 ml of chloroform was added to the solution, and incubated for 5 min. The samples were centrifuged for 5 min at 12,000*g* and 4 °C, and the aqueous phase from the phasemaker tube was transferred to a new tube. A 0.5 ml isopropanol and 10 µg glycogen were added and mixed by pipetting, and the resulting solution was incubated for 10 min at room temperature. The samples were then centrifuged. RNA was then purified using 75% ice-cold ethanol and dissolved in 350 µl RLT solution from RNeasy Mini Kit (QIAGEN GmbH, Hilden, Germany) and 3.5 µl of β-mercaptoethanol (Sigma-Aldrich) were added to the tube and mixed by pipetting. The solution was transferred to a gDNA eliminator column from RNeasy Mini Kit and centrifuged for 30 s at 8000*g* at room temperature. The rest of the experiment was continued according to manufacturer’s protocol. RNA concentrations and the purity were measured by a spectrophotometer (DeNovix, Wilmington, USA). Samples were used for cDNA synthesis^21,22^.

### RNA isolation from paw tissue cells

2 × 10^6^ cells from a single paw were used for each sample, and 350 µl RLT solution from RNeasy Mini Kit was added. RNA extraction from cells was performed using RNeasy Mini Kit according to the manufacturer’s protocol. RNA concentrations and the purity were assessed as previously described^21^ and samples were used for cDNA synthesis.

### Quantitative reverse transcriptase polymerase chain reaction (qRT-PCR)

The IV VILO Master Mix with ezDNase enzyme (Thermo Fischer Scientific, Waltham, MA, USA) was used to reverse transcribe RNA to cDNA according to manufacturer’s protocol. The qPCR was performed in duplicates using the TaqMan Fast Advanced Master Mix and TaqMan gene expression assays (Suppl. Table 1) (Thermo Fischer Scientific, Waltham, MA, USA) according to the manufacturer’s instructions. The additional no-RT controls were run to test for impurities or genomic DNA contaminations. The amplification was carried out for 40 cycles and relative expression levels were normalized using GAPDH as an endogenous reference gene, and calculated with the 2^−ΔΔCt^ method^21,23^.

### Statistical analyses

Eight rats or samples were used per group. Statistical analyses were performed using GraphPad Prism software (GraphPad Software Inc., San Diego, USA). Normality of data distribution was assessed by D’Agostino-Pearson omnibus normality test. Outliers were calculated using Grubb’s test and N = 1 was excluded from each of the following data sets: Fig. 5: TNF vehicle, IL-1β diclofenac, IL-6 diclofenac. Two sample comparisons to assess the effects of DCF compared to control on tissue pH and on gene expression, were assessed by a two-tailed unpaired t-test for normally distributed data, and by Mann Whitney test for non-normally distributed data. Two-tailed paired t-test was used to assess the pH between inflamed and non-inflamed paws of the same rat. Two-way repeated measures (RM) analysis of variance (ANOVA) with Bonferroni’s multiple comparison test was used to evaluate the effects of DCF on PPT and PV (vehicle vs. diclofenac treatment). One-way RM ANOVA with Bonferroni’s multiple comparison test was used to evaluate the effects of NFEPP and fentanyl on PPT over a time-course. Two-tailed unpaired t-test was used to evaluate the effects of PPT elevations produced by the most effective NFEPP and fentanyl dose in inflamed paws at 5 min. The data are expressed as individual data points and/or means ± SEM. Differences were considered significant at values of *P* < 0.05.

## Supplementary Information

Supplementary Table 1.

## Data Availability

All data generated or analysed during this study are included in this published article and its supplementary information files.
