# Supplementary material for: Amelioration of injury-induced tissue acidosis by a nonsteroidal analgesic attenuates antinociceptive effects of the pH-dependent opioid agonist NFEPP
Source: Sci Rep. 2022 Sep 7;12:15172. doi: 10.1038/s41598-022-19568-9 (PMC9452500; doi:10.1038/s41598-022-19568-9)
Supplement: Supplementary file 1 — Supplementary Table 1. [file 41598_2022_19568_MOESM1_ESM.pdf]

Supplementary Table 1

TaqMan gene expression assays used in qPCR

Oprm1 (Rn01430371_m1) for mu- opioid receptor (MOR)
Oprd1 (Rn00561699_m1) for delta-opioid receptor (DOR)
Oprk1 (Rn00567737_m1) for kappa-opioid receptor (KOR)
Il1b (Rn00580432_m1) for interleukin-1β (IL-1β)
Il4 (Rn01456866_m1) for IL-4
Il6 (Rn01410330_m1) for IL-6
LOC103694380 (Rn01525859_g1) for tumor necrosis factor-α (TNF-α)
NGF (Rn01533872_m1) for nerve growth factor (NGF)
Gapdh (Rn01775763_g1) for glycerinaldehyde-3-phosphate-dehydrogenase (GAPDH)
